# Combined Femoral Osteotomy and Contralateral Hip Arthroplasty to Correct Limb Alignment and Arthritis in a Scoliosis and Polio Patient: A Case Report

**DOI:** 10.7759/cureus.39010

**Published:** 2023-05-14

**Authors:** Ayush Balaji, Akira Toga, Aman Sanghai, Hammad Chishti, Luke Turner, Shojiro Katoh

**Affiliations:** 1 Hull York Medical School, University of York, York, GBR; 2 Department of Orthopaedic Surgery, Edogawa Hospital, Tokyo, JPN

**Keywords:** leg length inequality, distal femoral osteotomy, knee, hip, polio, arthritis, coronal limb alignment, scoliosis, total hip arthroplasty, osteoarthritis

## Abstract

We describe the first case of a 62-year-old female symptomatic patient with multiple comorbidities presenting with coronal limb malalignment due to scoliosis and osteoarthritis who underwent a combined total hip arthroplasty and biplane opening wedge osteotomy of the distal femur in one procedure. It is essential to realize that in patients who present with multiple comorbidities, combining different established procedures should be considered as a therapeutic option. We detail the operative procedure, preoperative considerations, and postoperative rehabilitation. By reviewing the literature on operative techniques, we highlight how our findings can be applied to similar cases with multiple comorbidities. Our report underscores the importance of considering combined procedures as a viable therapeutic option for patients with complex medical histories.

## Introduction

Coronal limb alignment is defined as the hip-knee-ankle axis that contributes to postural stability and mobility of the lower limb. Deviations from the normal alignment significantly impact the quality of life our patients face daily through limitations in activity, pain, and the ability to maintain healthy lifestyles. It can also play a critical role in the surgical management of conditions such as osteoarthritis and injury healing [1]. 

## Case presentation

**Figure 1:**
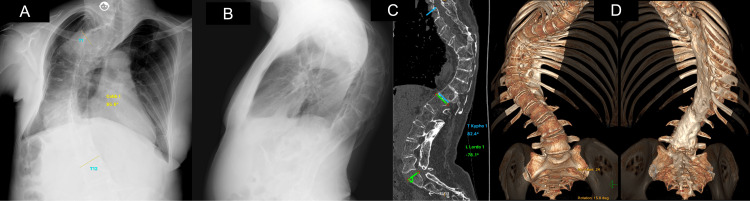
Imaging of the patient's spine (A) Anteroposterior Radiograph depicting scoliosis severity. (B) Lateral Radiograph depicting kyphosis severity (C, D) 3-Dimensional Computed Tomography of Spine. Parameters to indicate the severity of scoliosis have been annotated.

We describe the case of a 62-year-old female patient who underwent distal femoral osteotomy (DFO) and total hip arthroplasty (THA) at one stage to improve coronal limb alignment. On arrival, the patient presented with severe pain in both legs, pain in the right hip, and disturbances in gait. The patient had a history of severe scoliosis that was previously operated on, polio infection, hepatitis B infection, hepatitis C infection, cholangiocarcinoma, and a right lobectomy of the liver. Physical examination of the patient and the history showed that while the patient had an unstable gait and weakness of muscles due to polio, there was no significantly strong instability or hyperextension. The patient was able to weight-bear prior to surgery; however, due to pain, this was with difficulty. Radiographic examination indicated rotation of the pelvic ischium and ilium and showed severe erosion of the right acetabulum and femoral head (Figure 1).

**Figure 2:**
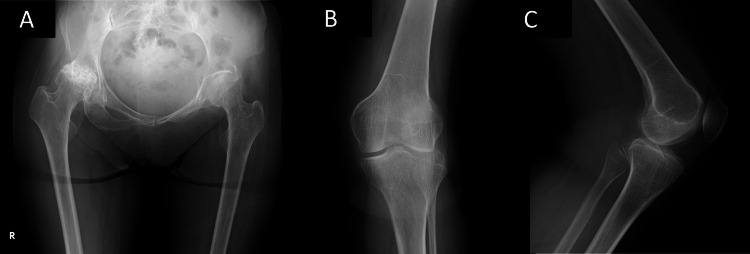
Preoperative radiographs Preoperative radiographs of the affected regions including the right hip and left knee joint. (A) Anteroposterior radiograph at the hip. (B) Anteroposterior radiographs of the knee C) lateral radiograph of the knee.

The obturator foramen on the left side is near invisible in an anteroposterior orientation with malformation of the iliac spine. Upon radiographic examination, the patient was diagnosed with pathological knock knees with severe valgus deformity and a functional deficit attributed to the difference in leg length of just above 2 cm (Figure 2).

She was also unable to walk for more than 200 meters without assistance because of right hip and knee pain. She also had a lift in her left shoe due to the difference in leg length between both legs.

The patient was initially followed up with a hyaluronic acid injection, celecoxib, and teriparatide treatment for osteoporosis; however, surgery was proposed due to continuing pain and symptoms. A clinical diagnosis of osteoporosis was made by her previous clinicians. Once the surgery was planned, teriparatide treatment was started to improve bone density and quality. A combined procedure was planned to relieve the symptoms of coronal malalignment and arthritis.

**Figure 3:**
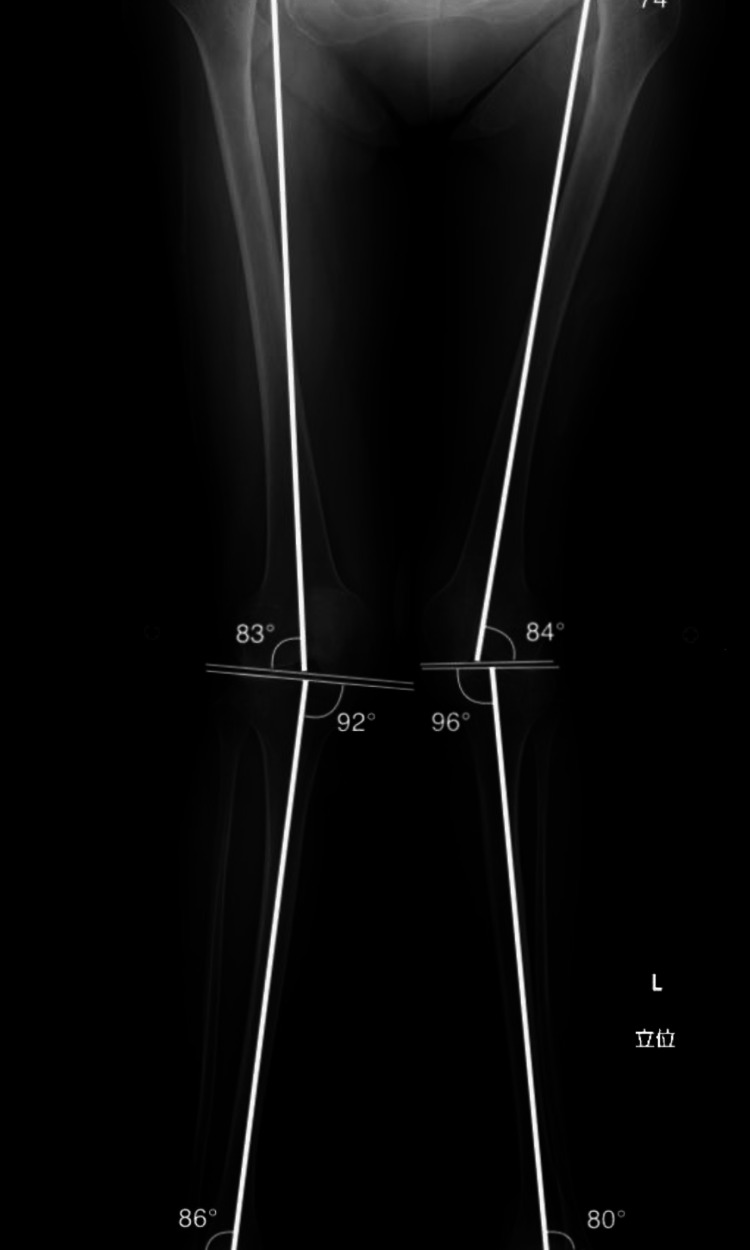
Preoperative measurement of alignment parameters Preoperative Radiograph of the entire lower limb with coronal alignment parameters.

The further preoperative study indicated excessive wear on the right hip joint and malalignment of the patient’s left knee. Femorotibial angle (FTA) measurements showed 159 degrees in the left leg, indicating severe valgus knock-knee deformity (Figure 3).

A distal femoral biplane open-wedge osteotomy was planned to increase the FTA as close as possible to the normal angle of approximately 176 degrees without causing a reduction in leg length and functional deficit [2]. The rationale for this procedure was attributed to the fact that the patient had a previous long spinal fusion procedure to correct her scoliosis resulting in rigidity and inability to accommodate further pelvic rotation. In her usual position, she had a strong anterior pelvic tilt. A combined procedure aided in preventing gait instability and coordination, thus reducing the risk of falls or further injury. 

**Figure 4:**
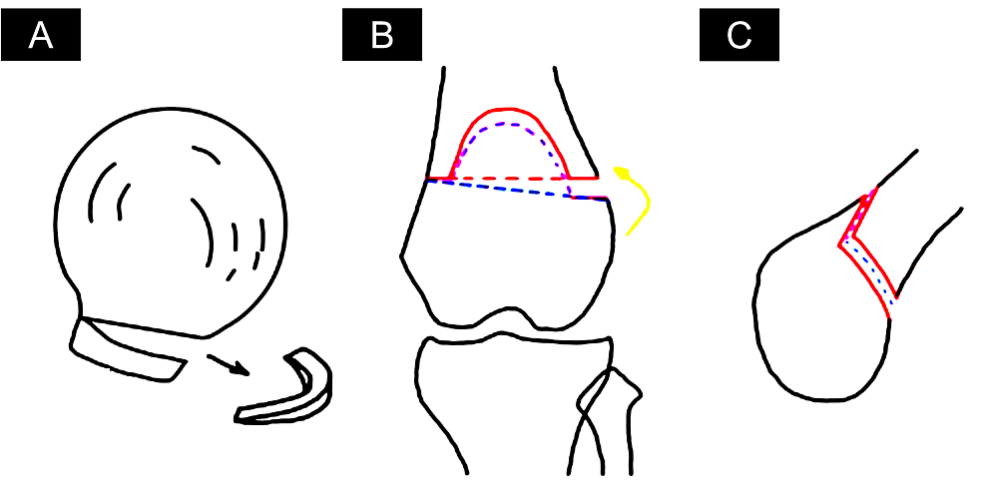
Diagrammatic rendering of surgical procedure Diagrams depicting operative procedure (A)  harvest of bone graft from the femoral head and (B) Opening wedge biplane distal femoral osteotomy plans with the purple dashed line depicting the vertical and posteriorly angulated cut towards the plane of the desired second lateral to medial osteotomy along the K-wire as shown by the blue dashed line. The orange arrow depicts the rotation of the osteotomy site.

First, a right THA was performed through a direct anterior approach. The femoral head was removed, and the acetabulum was reamed. A 50 mm hemispherical cup was set in the acetabulum with two 25.15 mm screws and a 28 mm liner. The joint capsule was dissected, and the femur was prepared for the insertion of a size 7.5 stem. An 8 mm offset was used for the implant neck to improve limb balance and bring the orientation closer to the Mikulicz line. This was also done to alleviate pain in the lateral right knee. A layered closure with a drain was done (Figure 4). 

**Figure 5:**
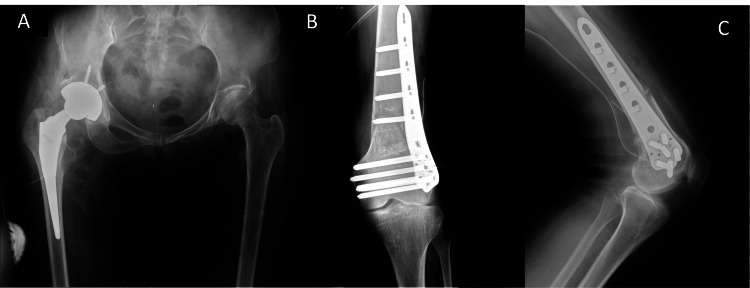
Postoperative radiographs Postoperative radiographs of the affected regions including the right hip and left knee joint. A) Anteroposterior radiograph at the hip. (B) Anteroposterior radiographs of the knee C) lateral radiograph of the knee.

There was a need to correct the valgus deformity in the left leg and increase leg length to improve balance with the right leg. Preoperative findings indicated that the left leg was 20 mm shorter than the right leg. A lateral biplane opening wedge osteotomy was performed on the left femur leading to an increase in the FTA to 168 degrees to address the preoperative complaints of leg length discrepancy and poor coronal alignment. A K-wire was inserted from the medial surface to guide the plane of the lateral osteotomy and establish the safe zones. An osteotomy was made from the front medial surface in a vertical and downwards-angulated direction toward the K-wire then a second osteotomy was made along the plane of the K-wire to create a lateral opening wedge. This allowed for optimal rotation of the femur to meet the correction parameters. This was determined through preoperative evaluation of the center of rotation angulation to preserve, if not increase, leg length. A graft from the contralateral hip was used as the wedge. The length of the left leg was increased by 20 mm, reducing the leg length discrepancy. A plate on the lateral side with locking screws was implanted to maintain the osteotomy’s patency to prevent the site’s instability and separation. It was a concern to us that the quadriceps muscle had a high degree of fatty degeneration due to polio, but the fixation was good (Figure 5).

**Figure 6:**
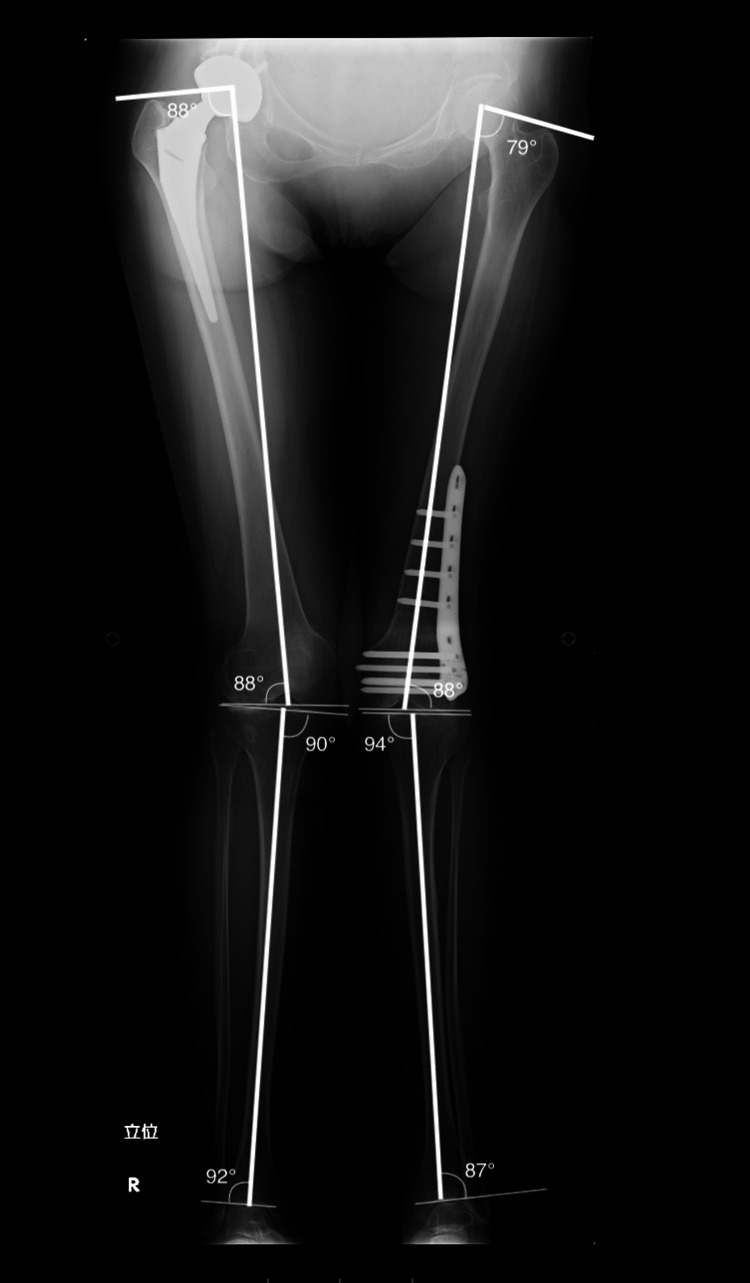
Postoperative Measurement of Alignment Parameters Postoperative Radiograph of the entire lower limb with Coronal alignment parameters.

The operative time was three hours and 18 minutes, and 300ml of blood loss was associated with the surgery. Post-operatively the parameters for coronal limb alignment were evaluated, and all showed significant improvements toward the normal reference range (Figure 6). 

The day after the operation, rehabilitation was started. During this immediate period, she reported that pain in her left lower leg was severe, and she had sharp pain around the right hip joint, likely attributed to postoperative pain however, there was an improvement seen throughout the postoperative period. There was a temporary limitation in her joint movement range and lower limb muscle weakness which made it difficult for her to continue with some of her activities of daily living, such as walking. We worked with ward nurses and rehabilitation staff to reduce her pain, improve her range of motion, improve muscle strength, and improve stability when performing basic movements such as walking and her other activities of daily living. This was done through both passive and active joint mobilization. Her range of motion gradually improved as time progressed. Hip flexion specifically, improved from 30 degrees preoperatively to 100 degrees during the rehabilitation period which was noted as significant as the patient had difficulty standing up from a seated position and also during prolonged sitting on the floor. Manual muscle testing (MMT) revealed a full range of motion against gravity for both left knee flexion and extension, grade 3, and a full range of motion with gravity eliminated of hip circumduction, grade 2, during the initial stages of rehabilitation similar to her preoperative condition. Weight-bearing with a semi-circular walker was used to support the patient during the initial postoperative period. Initially, a circular walker was used to assist the patient with walking then a transition was made to crutches within one-week post-operation. She was able to comfortably stand up from a seated position and was able to walk up to 300 meters without pain. Deep vein thrombosis mechanical prophylaxis was also started immediately postoperative due to the restricted mobility of the patient. This included the use of a peristaltic foot pump as well as stockings. D-dimer monitoring and pharmacological prophylaxis with edoxaban were also continued for approximately two weeks. The rehabilitation timeline progressed over a span of two weeks. A lower-extremity functional assessment score comparison between the pre-and post-operative is shown between Table 1 and Table 2. 

**Table 1:** Pre-operative Lower Extremity Functional Scale (LEFS) Pre-operative Lower Extremity Functional Scale (LEFS) Score for the Patient to assess functional status

Activities	Extreme Difficulty or Unable to Perform Activity	Quite A Bit of Difficulty	Moderate Difficulty	A Little Bit of Dificulty	No Difficulty
Any of your Usual Work, Housework, or School Activities	0	1	2	3	4
Your Usual Hobbies, Recreational, or Sporting Activities	0	1	2	3	4
Getting into or out of the bath	0	1	2	3	4
Walking between Rooms	0	1	2	3	4
Putting on Shoes or Socks	0	1	2	3	4
Squatting	0	1	2	3	4
Lifting an object, like a bag of groceries of the floor	0	1	2	3	4
Performing Light Activities around your home	0	1	2	3	4
Performing Heaving Activities around your home	0	1	2	3	4
Getting into or out of a car	0	1	2	3	4
Walking 2 Blocks	0	1	2	3	4
Walking a mile	0	1	2	3	4
Going up or down 10 stairs	0	1	2	3	4
Standing for 1 hour	0	1	2	3	4
Sitting for 1 hour	0	1	2	3	4
Running on even ground	0	1	2	3	4
Running on uneven ground	0	1	2	3	4
Making Sharp Turns while running fast	0	1	2	3	4
Hopping	0	1	2	3	4
Rolling Over in Bed	0	1	2	3	4

**Table 2:** Post-operative Lower Extremity Functional Scale (LEFS) Score Post-operative Lower Extremity Functional Scale (LEFS) Score for the Patient to assess functional outcomes.

Activities	Extreme Difficulty or Unable to Perform Activity	Quite A Bit of Difficulty	Moderate Difficulty	A Little Bit of Dificulty	No Difficulty
Any of your Usual Work, Housework, or School Activities	0	1	2	3	4
Your Usual Hobbies, Recreational, or Sporting Activities	0	1	2	3	4
Getting into or out of the bath	0	1	2	3	4
Walking between Rooms	0	1	2	3	4
Putting on Shoes or Socks	0	1	2	3	4
Squatting	0	1	2	3	4
Lifting an object, like a bag of groceries of the floor	0	1	2	3	4
Performing Light Activities around your home	0	1	2	3	4
Performing Heaving Activities around your home	0	1	2	3	4
Getting into or out of a car	0	1	2	3	4
Walking 2 Blocks	0	1	2	3	4
Walking a mile	0	1	2	3	4
Going up or down 10 stairs	0	1	2	3	4
Standing for 1 hour	0	1	2	3	4
Sitting for 1 hour	0	1	2	3	4
Running on even ground	0	1	2	3	4
Running on uneven ground	0	1	2	3	4
Making Sharp Turns while running fast	0	1	2	3	4
Hopping	0	1	2	3	4
Rolling Over in Bed	0	1	2	3	4

In a four-year follow-up, the patient mentioned that her mobility significantly improved and she could carry out many of her activities of daily living. The coronal limb alignment was maintained with no significant changes. The THA implant and osteotomy site were both stable. There was a reduction in pain at the right hip and improved mobility compared to her preoperative state. 

## Discussion

Before conducting a DFO or THA, it is vital to contemplate certain preoperative factors, such as determining the level of the osteotomy, the measurement of the osteotomy angle based on hip-knee alignment, as well as choosing the correct osteotomy wedge size for the patient [3]. In our case, we assessed the parameters and compared them to the normal range reported [4]. In this case, an open wedge biplane osteotomy was performed at the distal femur, adding a bone graft between the two ends of the wedge incision, thereby preventing shortening [5, 6]. Due to the patient’s leg length discrepancy, we chose the open wedge approach, which was ideal for correcting their coronal limb alignment and improving leg length matching [7]. The biplane variant of the osteotomy was selected as, from our experience, we found it to be resistant to postoperative fracture and malrotation, as well as surgeon preference. In this particular case, we found that a key aspect was not just increasing the leg length of the patient but also having the ability to correct the limb axis during surgery. The biplane distal femoral osteotomy allowed us to correct the deformity through rotation and graft placement to better serve the patient's desired outcome while providing stability.

The procedure was done as one rather than two for various reasons. One was because the femoral head recovered from the THA could be used to create the graft for the lateral open wedge osteotomy, saving the patient from requiring a graft harvest procedure from another site. Another reason was the well-known risk of anesthetics in patients with a history of liver disease such as ours due to the known hepatotoxic effects of multiple anesthetics [8]. These factors, combined and patient preference, directed us to perform one procedure under one round of anesthesia. Monitoring and preoperative screening by consultant anesthetists, internal medicine consultants, and the operative team were conducted to ensure safe practice. 

This is the first report of a case where THA was done with a contralateral DFO in a single procedure and with this specific technique. When attempting to treat cases such as these without established management guidelines, it is essential to use various levels of evidence, plan for any complications, and prepare rehabilitation protocols. This should be managed through a multidisciplinary approach making sure to include the patient and family for home care post-discharge to ensure good outcomes. Challenges of a single procedure on multiple operative sites include difficulties in operative prepping and the management of multiple wound sites. Rehabilitation time may be increased as well. However, due to a history of polio infection, the pain sensation in the patient’s left leg was already reduced, allowing for a typical rehabilitation timeline. Post-operatively, different goals for each stage of the rehabilitation process should be employed to combat complications correspondingly [9 - 12]. Due to a history of polio and scoliosis, the phases were adapted to suit the paralysis of muscles surrounding the left knee joint and mobility issues. The autologous bone graft from the contralateral femoral head was a strategy employed due to the superior mechanical property and the improved healing capability of autologous bone grafts influenced our choice in its use. Reinforcement with the locking screws and plate added further stability [13]. 

It is essential to realize that in patients who present with multiple comorbidities, our aim should be to fix as many issues as possible that cause symptoms or interfere with their quality of life. This may not lead to the complete correction of a deformity; however, the essential factor to consider is whether the patient positively impacts the quality of life. 

## Conclusions

It is important to realize that various factors can influence the inclusion of patients for procedures that deviate from the standard ones performed. Physician experience and expertise, as well as the operative team’s familiarity with the operative plan, is vital in managing patients undergoing such procedures. Patients with comorbidities should be evaluated on a case-by-case basis to determine the optimal pathway for their care. A multidisciplinary approach is paramount in their management. Combined procedures should be considered based on the value they propose to the patient’s quality of life and the recovery protocols in place to ensure adequate rehabilitation for the patient to return to their activities of daily living. 
